# The mediating role of maladaptive perfectionism in the relationship between childhood trauma and depression

**DOI:** 10.1038/s41598-025-03783-1

**Published:** 2025-05-25

**Authors:** Sylwia Michałowska, Magdalena Chęć, Piotr Podwalski

**Affiliations:** 1 Department of Clinical Psychology, Institute of Psychology, University of Szczecin, Krakowska St. 69, Szczecin, 71-017 Poland; 2 Department of Psychiatry, Pomeranian Medical University, Broniewskiego 26 Street, Szczecin, 71-460 Poland

**Keywords:** Childhood trauma, Perfectionism, Depression, Neglect, Violence, Sexual abuse, Psychology, Human behaviour

## Abstract

Childhood trauma resulting from violence, abuse, and neglect has long-term effects on health and is linked to the development of diseases and mental disorders, including depression. It turns out that traumatic experiences in childhood can also foster traits of perfectionism, whose maladaptive form may further increase the risk of developing depression. The aim of this study was to assess the role of perfectionism in the relationship between childhood trauma and depression in adulthood. The analysis involved 308 participants (73 with depression and 235 healthy controls). The study used questionnaires assessing childhood traumatic experiences (MACE-58) and perfectionism levels (KPAD). The results showed that individuals with depression exhibited higher maladaptive perfectionism and a greater severity of trauma, particularly physical violence and sexual abuse. Traumatic experiences, especially sexual abuse and physical violence from peers, were significant predictors of depression. It was found that maladaptive perfectionism mediated the relationship between trauma and depression, eliminating the direct link in the case of some traumas, such as sexual abuse. The study highlights the significant role of maladaptive perfectionism in the development of depression in individuals who experienced traumatic events in childhood, suggesting that interventions aimed at reducing this type of perfectionism may positively impact depression treatment.

## Introduction

Childhood trauma (CT) can be defined as a child’s experience of an event or series of events that are physically and/or emotionally harmful, painful and distressing and often result in negative physical, emotional and social consequences for the child, as well as affect the child’s psychological wellbeing^1–3^. According to the definition of the World Health Organisation (WHO)^4^, CT is “all forms of physical and/or emotional ill-treatment, sexual abuse, neglect or negligent treatment or commercial or other exploitation, resulting in actual or potential harm to the child’s health, survival, development or dignity in the context of a relationship of responsibility, trust or power”^4^. Therefore, any type of physical or mental (emotional) abuse, sexual abuse, neglect (physical or emotional) experienced by a person under the age of 18 can be considered CT^5^. Abuse against a child or teenager may be perpetrated by a variety of people, but the perpetrators are often people from the young person’s immediate surroundings – family and peers^6^. CT may also happen as a result of observing violence against other people, especially persons close to the child^7^. Different types of CT often form a complex pattern of co-occurrence, and the presence of negative experiences in childhood increases the odds of more subsequent trauma^8^. The negative effects of childhood trauma are cumulative, supporting the link between both the amount and type of trauma experienced and the number and severity of resulting health problems^9^.

This understanding of childhood trauma can be linked to type II trauma distinguished by Terr^10^, which, unlike type I trauma (e.g. car accident), lasts for a long time and reoccurs in childhood, shaping slowly, becoming severe and causing a broad negative impact on health. According to Terr, type II trauma—a form of trauma characterized by repeated or prolonged exposure to traumatic events—can occur during childhood or at any stage of life, and is considered a subtype of childhood trauma when experienced early in life^10^.

Childhood trauma is one of the main problems in today’s world. Despite social campaigns run in many countries and widespread systemic changes, it is still sadly a common occurrence in both the general and clinical population^11^. It should be noted that data on the number and severity of CT is most likely underestimated, as many people hide or do not report abuse in fear of social consequences. Nevertheless, it is considered that globally even 1 billion children aged 2–17 experience physical, emotional, sexual abuse or neglect every year^11,12^. According to a 2023 report by the nationwide Empowering Children Foundation, which deals with abuse prevention and support for affected children, even 66% of Polish children and adolescents experience abuse from peers, 32% are abused by adult relatives, 26% have been sexually abused without physical contact and 23% have been emotionally neglected^13^.

According to a meta-analysis by Hughes et al.^14^, CT is a factor that significantly affects a person’s health over the course of their lifetime – increasing the risk of developing both somatic diseases (such as diabetes, respiratory and cardiovascular diseases) and psychiatric disorders (including depression). In adulthood, CT is also associated with a worse prognosis and lesser response to treatment of diseases and disorders, in the case of affective disorders, with their earlier onset, more severe and recurrent symptoms, and more difficult and less effective pharmacological and psychotherapeutic treatment^7,15–18^.

When considering the relationship between childhood trauma and future psychological disorders, it can be explained with the classical developmental psychopathology theory, where CT is an important factor of psychological distress in the course of human development^19^. Biological models explain this relationship as follows: experiencing childhood trauma can permanently change an individual’s responses to stress, causing mental health disorders in adulthood. Chronic activation of the hypothalamic-pituitary-adrenal axis (HPA) can lead to persistent secretion of stress hormones, which trigger dysregulation in the stress axes and can lead to health problems^20,21^. Research to date suggests correlations between CT and disorders such as post-traumatic stress disorder (PTSD)^22,23^, anxiety disorders^5,24^, depression^25–27^, sleep disorders^28^, psychosomatic disorders^29^, eating disorders^30^, alcohol and substance abuse^31^, psychotic disorders including schizophrenia^32,33^ and personality disorders, especially borderline personality disorder^34^. Some researchers have examined the magnitude of the impact of different forms of trauma with the occurrence of a particular type of disorder in adulthood. Physical and emotional abuse have been linked to PTSD, depression and anxiety disorders, and physical abuse also to ADHD. Sexual abuse was most often associated with psychosomatic disorders and PTSD. Emotional neglect in childhood was associated with depression, eating disorders and substance abuse, while physical neglect was associated with depression and schizophrenia^35,36^. Research on the role of gender in the relationship between trauma and its subsequent negative impact on mental health is inconclusive and culturally linked^36^. It suggests that post-traumatic occurrence of depression and PTSD affects women more often than men, while trauma affects men’s psychological wellbeing to a greater extent.

## Trauma and depression

Experiencing negative and stressful events early in life is an important element in the development of depression in adulthood^37^. According to most recent knowledge, being exposed to CT increases the risk of depression by 3–4 times^14,38^.

Depressive disorder is a common mental disorder affecting more women (6%) than men (4%). According to the American Psychological Association, it is the most common mental illness^39^, affecting around 280 million people worldwide. Based on WHO statistics^40^, approximately 5% of the adult population suffers from depression. According to the Global Burden of Disease Study 2016^41^, which assessed the prevalence, incidence and years lived with disability in 195 countries, severe depression is among the top five causes of disability, alongside back pain, migraine, hearing loss and anaemia.

Depression is a mood disorder mainly characterised by a low mood prevailing over a long period of time and loss of enjoyment or interest in previous activities. The fifth edition of the Diagnostic and Statistical Manual of Mental Disorders (DSM-5)^42^ classifies depressive disorders as a group of specific diagnostic entities, including disruptive mood dysregulation disorder, major depressive disorder, persistent depressive disorder (dysthymia), premenstrual dysphoric disorder, and depressive disorder due to another medical condition. Regardless of the specific type, these disorders are characterized by a persistent feeling of sadness, emptiness, or irritability, accompanied by somatic and cognitive changes that significantly impair daily functioning and affect both personal and professional aspects of life^42^. There is a number of factors, including genetic, physical and socio-emotional, that contribute to the development of depression^43^, and CT is one of its strongest predictors at any age^44–46^. Research suggests that the development of depression is connected to the type and severity of CT^47^ – the more severe the abuse and neglect, the greater the risk of developing the disorder in adulthood^48^. Symptoms of severe depression are most strongly correlated with emotional abuse^15,18,31,47^, followed by neglect^15,45^ and sexual abuse^48,49^. In addition to CT type, depression can also be influenced by gender, age at the time of the abuse, as well as the extent and duration of the abuse^50^. In order to better understand the links between CT and depression, some researchers focus on finding mediators and moderators of these relationships. Few studies reveal the indirect role of physical factors. One study showed a mediating role of BMI, insomnia, and cigarette smoking in 3 to 26% of the correlations between health-related factors (such as physical activity, stress, and diet) and mental health outcomes (such as depression and anxiety)^51^. The mediators include psychological factors such as difficulties with emotional regulation, attachment, attribution style and jealousy. A study conducted by Zhang et al.^52^ showed an incomplete mediating effect of adaptive and maladaptive emotional regulation strategies, where the indirect effect of childhood trauma on depression through adaptive strategies accounted for 20.9% of the total effect and through maladaptive strategies for 16.3% of the total effect. In another moderation-mediation study among students, stress perception and neuroticism were chain mediators between childhood trauma and depression, and adaptive cognitive emotional regulation strategies played a moderating role in the trauma-neuroticism-depression relationship^53^.

### Role of perfectionism in the development of depression in people with early-childhood trauma

Perfectionism is described as a personality trait associated with setting high standards for oneself, focusing excessively on mistakes and feeling the need to be seen as perfect^54^. Frost et al.^54^ distinguished 6 dimensions of this trait: Concern over Mistakes, Personal Standards, Parental Expectations, Parental Criticism, Doubts about Actions, and Organization. Individuals with a perfectionist disposition may display positive perfectionism, called adaptive perfectionism, or negative perfectionism, i.e. maladaptive perfectionism^55^. Individuals representing adaptive perfectionism show high personal standards and a need for good organisation, which is linked to the pursuit of set goals. However, if an individual focuses on high expectations, perceived parental criticism, and mistakes, they may exhibit characteristics of maladaptive perfectionism^56^, which increases the risk of depression^57–59^ as it is associated with experiencing undesirable emotions^60^.

Theoretical models focusing on the etiology of perfectionism offer important findings. The maladaptive form of perfectionism can be linked to negative life events^61^. According to the Perfectionism Social Disconnection Model (PSDM)^62^ and the Social Reaction Model of Perfectionism (SRMP)^63^, difficult experiences or abuse in childhood are important vulnerability factors favouring the development of perfectionism. According to the assumptions of the PSDM^62^ and SRMP^63^, perfectionism develops partly in response to adverse parental behaviour and traumatic childhood experiences, which were linked with the feelings of despair, shame, powerlessness and insecurity. These models assume that after such experiences the individual strives for perfection in order to secure love and acceptance, regain a sense of control and reduce feelings of despair, shame and helplessness and thus avoid further abuse^62,63^. Numerous studies have confirmed that adverse parental behaviours, such as excessive control, strictness, neglect and conditional approval, are associated with the development of perfectionism^64–67^. A factor that sustains the recollection of traumatic experiences is ruminations, characteristic of both perfectionism^68,69^ and depression^70,71^.

Other studies suggest that the experience of childhood abuse and neglect are important predictors of perfectionism and that maladaptive perfectionism that emerges as a consequence of childhood trauma is associated with the experience of more severe stress^72^. The more stressful events a person has experienced, the more susceptible they are to developing depressive symptoms^73^. Maladaptive perfectionism based on perfectionist fears also helps predict the occurrence of so-called major depression^74^. Taking into account how destructively traumatic childhood experiences affect attachment style in adulthood^2^, it is also worth considering studies that have found that perfectionism mediates the relationship between insecure attachment and depression in adults^75,76^. Research on adolescents has also found significant links between insecure attachment, perfectionism and depression^77^. Empirical evidence also supports the idea that negative life events strengthen the correlation between perfectionism and depression^78,79^.

Given the prevalence of childhood trauma and its negative effects, as well as the lack of sufficient research confirming the role of the perfectionism mechanism in the emergence of psychopathology, it was decided to explore the problem, extending contemporary knowledge on risk factors and protective factors in the development of depression after early-childhood trauma. Based on contemporary theoretical knowledge and research findings, the main objective of this analysis is to verify whether perfectionism, both adaptive and maladaptive, is a mediator in the relationship between adverse childhood experiences and the occurrence of depression in adults.

## Methods

All experiments were performed with approval of the Ethics Committee for Research Projects of the Institute of Psychology at the University of Szczecin in Poland. All experimental procedures and protocols involving human participants/tissues were conducted in full accordance with relevant institutional, national, and international guidelines, including the Declaration of Helsinki and the European Union General Data Protection Regulation (GDPR).

Informed consent was obtained from all individual participants included in the study.

## Participants

Participants in the study were individuals diagnosed with depression by a qualified specialist, a psychiatrist, and healthy people forming the control group. Inclusion criteria for the study included being over 18 and having good oral and written Polish language skills, adequate cognitive ability and mental health to complete the entire questionnaire survey. The selection for the study was purposive - random. The clinical group included patients hospitalised at the Day Unit and at the 24-hour Wards in the north-west of Poland. A total of 308 participants took part in the study, with 187 women (60.8%) and 121 men (39.2%). The clinical group comprised 73 people (23.7%) − 45 women (61,6%) and 28 men (38,4%). The non-clinical group comprised 235 people (76.3%) − 142 women (60,4%) and 93 men (39,6%). There was no significant association between gender and group membership (*p* = 0.852). In the clinical group, the mean age was 42.1 years (SD = 16.2), while in the non-clinical group it was 40.8 years (SD = 14.2).

In clinical group 27,4% had higher education, 41,1% lived in cities with 100,000–500,000 inhabitants, and 32,9% were in a relationship. In non-clinical group 41,7% had higher education, 50,6% lived in cities with 100,000–500,000 inhabitants, and 47,7 were in a relationship.

In the current study, depression was treated as a categorical variable (presence vs. absence in the clinical and non-clinical groups). For the clinical group, participants were hospitalized in psychiatric wards, and depression was their medical diagnosis. In the non-clinical group, participants reported having received a diagnosis of depression from a psychiatrist, as indicated in the sociodemographic survey; however, these individuals were not hospitalized nor currently exhibiting symptoms of depression. The inclusion of participants in the non-clinical group was based on their self-reported history of depression diagnosis.

## Procedure

The research was carried out using the paper-pencil method. This included the use of a questionnaire designed to investigate childhood traumatic experiences, the MACE-X (Maltreatment and Abuse Chronology of Exposure) by Teicher and Parigger^80^ in a Polish adaptation (MACE-58 (PL))^81^. The analyses also used the Polish Questionnaire of Adaptive and Maladaptive Perfectionism (KPAD) by Szczucka^82^ and an original questionnaire to collect sociodemographic data.

Participants from the clinical groups were individuals who were hospitalised at psychiatric wards, and they completed the questionnaires in group settings within therapeutic rooms, under the supervision of a researcher. Each participant was supported by a psychologist, who served as their guide throughout the study.

The non-clinical group comprised individuals who voluntarily signed up for the study. These individuals were recruited using a snowball sampling method, where initial participants were encouraged to share information about the study with others in their social networks, thus expanding the pool of volunteers. Participants in this group also completed the questionnaires in the presence of a researcher, who served as a guide throughout the process.

Participation for all was voluntary. Each test sheet was labelled with information about the purpose of the study and included informed consent to participate in the study, information about the anonymity of the data, and about the possibility to opt out at any time without giving a reason. The sheets completed during the study were placed in paper envelopes and sealed until the researchers opened them while working on coding the obtained data.

## Measures

The MACE-58 (PL)^81^ was used to assess whether traumatic experiences occurred in the subjects’ childhood and what their nature, time of occurrence and duration was. The Polish version of the tool contains 58 items, covering 10 types of neglect occurring during childhood and adolescence (up to the age of 18). The results are captured on the following scales: Parental verbal abuse (PVA), Emotional neglect (EN), Physical neglect (PN), Parental non-verbal emotional abuse (PNEVA), Parental physical maltreatment (PPA), Witnessed interpersonal violence to parents (WITP), Witnessing violence to siblings (WITS), Peer verbal abuse (PEERE), Peer physical bullying (PEERP), Childhood sexual abuse (SEXA). These ten subscales are derived from the original Maltreatment and Abuse Chronology of Exposure (MACE) scale developed by Teicher and Parigger^80^, and represent discrete categories of early adverse experiences, assessed retrospectively. Each subscale is designed to capture both the presence and developmental timing of a specific form of maltreatment or neglect between ages 1 and 18:

Parental Verbal Abuse (PVA): Involves exposure to hostile verbal behavior from caregivers, such as yelling, name-calling, blaming, and threats.

Emotional Neglect (EN): Reflects the absence of emotional responsiveness, affection, or support from caregivers.

Physical Neglect (PN): Involves failure to provide basic physical needs, including consistent food, shelter, clothing, hygiene, or medical care.

Parental Non-Verbal Emotional Abuse (PNEVA): Includes harmful non-verbal behaviors from caregivers, such as cold stares, dismissive gestures, or prolonged silent treatment.

Parental Physical Abuse (PPA): Covers physical aggression from caregivers, including slapping, punching, use of objects, or other forms of physical harm.

Witnessed Interpersonal Violence Toward Parents (WITP): Involves witnessing violence or threats between caregivers or between a caregiver and another adult.

Witnessed Violence Toward Siblings (WITS): Captures instances where the participant observed acts of physical aggression directed at siblings.

Peer Verbal Abuse (PEERE): Reflects experiences of verbal victimization by peers, including teasing, mocking, or humiliation.

Peer Physical Bullying (PEERP): Encompasses being physically attacked or threatened by peers, such as pushing, hitting, or intimidation.

Childhood Sexual Abuse (SEXA): Covers any unwanted sexual experiences, including inappropriate touching, exposure, or coercion.

The MACE framework emphasizes a chronological perspective, allowing for detailed assessment of the onset, duration, and accumulation of maltreatment across different developmental stages. This structure enables computation of total exposure scores, duration indices, and multiplicity (i.e., the number of different maltreatment types experienced), offering a nuanced understanding of childhood adversity^80,81^.

The following summed MACE scores were used in the statistical analysis:

1. Ten subscale scores, summing the items with positive responses for each subscale, with each subscale score matched on a scale from 0 to 10 points.
2. The MACE summation score (MACE SUM), which is the sum of the scores for the 10 subscales (range 0–100).
3. Multiplicity score (MACE MULTI) for the number of different types of maltreatment (range 0–10), where each type must be reported above the clinical cut-off level calculated.
4. Total duration score (MACE Duration), which is the number of years with exposure to at least one type of maltreatment above the clinical cut-off level calculated (range 0–18).
5. Total summation score by duration (MACE Sum by Duration), obtained by first calculating the summary score for each age level (1–18) and summing the scores for the 10 subscales in that age category, and then summing these scores for the 18 age groups and dividing the score by 18 to obtain a 0–100 range scale.

The tool in the Polish adaptation is characterised by convergent and predictive accuracy. The reliability of the tool based on the Kuder-Richardson index (KR20) for the individual scales of the tool is: Parental verbal abuse (PVA)- 0.827, Emotional neglect (EN)- 0.781, Physical neglect (PN)- 0.569, Parental non-verbal emotional abuse (PNEVA)- 0.648, Parental physical maltreatment (PPA)- 0.782, Witnessed interpersonal violence to parents (WITP)- 0.791, Witnessing violence to siblings (WITS)- 0.718, Peer verbal abuse (PEERE)- 0.795, Peer physical bullying (PEERP)- 0.647, Childhood sexual abuse (SEXA)- 0.867.

Perfectionism in the subjects was determined using the Questionnaire of Adaptive and Maladaptive Perfectionism^82^ (KPAD), which was developed on the basis of Frost’s theory of perfectionism^54^. The tool consists of 35 test items categorised into two scales: Adaptive Perfectionism and Maladaptive Perfectionism. The respondent answers using a 7-point Likert scale (from 1- strongly disagree to 7- strongly agree). The higher a score on each of the subscales, the higher the level of perfectionism of a particular type that individual exhibits. The original reliability for the Maladaptive Perfectionism scale is α = 0.953 and for the Adaptive Perfectionism scale α = 0.896. Cronbach’s alpha in the study was 0.896 for the Adaptive Perfectionism scale and 0.952 for the Maladaptive Perfectionism scale.

### Aims

Based on the literature review and the adopted theory, the following hypotheses were formulated:

H1: There is a relationship between childhood traumatic experiences and the risk of depression later in life.

H2: Perfectionism, particularly its maladaptive form, may mediate relationship between childhood traumatic experiences and the risk of depression in later life.

H3: It is not expected that adaptive perfectionism will mediate the relationship between childhood trauma and depression.

## Analysis

In order to compare the clinical and non-clinical group in terms of the variables analysed, an analysis was performed using the Mann-Whitney *U* test. A logistic regression analysis was performed to identify predictors of depression. The collinearity of the predictors was controlled with VIF < 3. In order to test the mediating role of perfectionism for the relationship between early childhood trauma and the onset of depression, analyses were conducted using A. Hayes PROCESS macro (version 4.2, model 4). Analyses were performed using IBM SPSS Statistics v. 29.0. The adopted level of significance was α = 0.05. The significance of mediating effects was estimated using the bootstrap method for sampling of 5000.

## Results

### Early childhood trauma experiences and the incidence of depression

The subjects were divided into two groups – those diagnosed with depression (clinical group) and those not diagnosed with this disorder (non-clinical group). Table 9 compares the clinical and non-clinical group in terms of variables associated with early childhood trauma and perfectionism severity. The analysis showed higher levels of physical abuse by peers and sexual abuse in the clinical group compared to the non-clinical group (weak effect). Both of these differences were also noted for multiplicity (*score for the number of different types of abuse (range 0–10)*,* where each type must be reported above the clinical cut-off level calculated with reference to the clinical cut-off values in the CTQ questionnaire used for the MACE-58(PL) validity test*) of these scales – higher severity occurred in the clinical group. The clinical group had higher levels of maladaptive perfectionism compared to the non-clinical group (weak effect). Adaptive perfectionism was higher in the non-clinical group than in the clinical group (weak effect).

**Table 1:** *Comparison of clinical and non-clinical group in terms of early childhood trauma and perfectionism* non-clinical (*n* = 235) clinical (*n* = 73).

Dependent variable	average rank	M	SD	average rank	M	SD	Z	*p*	*R*
Verbal abuse by parents	154.30	4.44	3.60	155.16	4.52	3.93	−0.07	0.94	< 0.01
Emotional neglect	150.56	4.55	2.81	167.17	5.10	2.55	−1.40	0.16	0.08
Physical neglect	148.87	4.43	2.17	172.62	5.11	2.64	−2.04	0.04	0.12
Non-verbal and emotional abuse by parents	154.94	2.79	2.54	153.08	2.81	2.75	−0.16	0.87	< 0.01
Physical abuse by parents	152.22	2.99	3.07	161.83	3.64	3.85	−0.83	0.40	0.05
Witnessing Violence Towards Parents	152.64	2.88	3.48	160.49	3.12	3.33	−0.71	0.48	0.04
Witnessing Violence To Siblings	150.32	1.05	1.97	163.64	1.62	2.66	−1.40	0.16	0.08
Emotional abuse by peers	152.06	3.69	3.57	162.34	4.03	3.41	−0.89	0.37	0.05
Physical abuse by peers	149.79	0.96	2.01	169.66	1.64	2.54	−2.13	0.03	0.12
Sexual abuse	148.72	0.74	1.72	173.12	1.71	2.92	−2.63	0.01	0.15
Overall MACE score	150.42	28.58	17.86	163.33	33.30	23.00	−1.09	0.28	0.06
MULTI_PVA	152.77	0.46	0.50	160.05	0.51	0.50	−0.71	0.48	0.04
MULTI_EN	150.90	0.72	0.45	166.08	0.82	0.39	−1.69	0.09	0.10
MULTI_PN	152.45	0.64	0.48	161.09	0.70	0.46	−0.88	0.38	0.05
MULTI_PNVEA	153.93	0.46	0.50	156.34	0.48	0.50	−0.23	0.81	0.01
MULTI_PPA	153.19	0.43	0.50	158.73	0.47	0.50	−0.54	0.59	0.03
MULTI_WITP	154.18	0.18	0.39	155.53	0.19	0.40	−0.17	0.87	< 0.01
MULTI_WITS	151.14	0.15	0.36	161.03	0.22	0.42	−1.28	0.20	0.07
MULTI_PEERE	156.10	0.40	0.49	149.35	0.36	0.48	−0.67	0.50	0.04
Multi_PEERP	149.95	0.08	0.27	169.14	0.21	0.41	−2.96	0.00	0.17
MULTI_SEXA	149.54	0.23	0.42	170.46	0.37	0.49	−2.29	0.02	0.13
MACE_MULTI	150.17	3.78	2.50	164.12	4.32	2.93	−1.18	0.24	0.07
Summary score by duration	150.04	12.70	9.18	166.68	15.36	11.41	−1.40	0.16	0.08
Training duration	152.06	12.33	6.72	160.23	13.12	6.51	−0.73	0.47	0.04
Neglect_sum	149.23	8.97	4.58	171.45	10.21	4.89	−1.86	0.06	0.11
Abuse_sum	151.38	19.61	15.06	160.25	23.09	19.70	−0.75	0.45	0.04
Maladaptive_perfectionism	142.85	3.52	1.33	177.89	4.08	1.51	−2.95	**0.00**	0.17
Adaptive_perfectionism	158.73	4.64	1.14	125.49	4.14	1.34	−2.8	**0.01**	0.16

*Comment.* Bolded rows indicate statistical significance at *p* < 0.05 level.

Source: own study.

### Models explaining the occurrence of depression based on early childhood trauma

The first model (Table 2) shows the results of a logistic regression analysis for a model explaining the occurrence of depression based on the severity of the type of early childhood trauma. The model was fit to the data χ^2^(10) = 21.40; *p* = 0.018 and explained a total of 6.8% of the variation in the group, (R^2^_Cox and Snell_ = 0.068). Sexual abuse was found to be the only significant predictor of depression – as the severity of sexual abuse increased by 1 point, the odds of depression increased by 21%. Other types of trauma were not significantly associated with the occurrence of depression.

**Table 2:** Logistic regression coefficients for a model explaining the incidence of depression based on type of early childhood trauma.

Type of trauma	B	SE	Wald	*p*	OR	95% CI for OR	
						LL	UL
Verbal abuse by parents	−0.10	0.07	1.95	0.162	0.91	0.79	1.04
Emotional neglect	0.01	0.09	0.02	0.890	1.01	0.85	1.20
Physical neglect	0.11	0.09	1.55	0.213	1.12	0.94	1.34
Non-verbal and emotional abuse by parents	−0.07	0.08	0.76	0.383	0.93	0.79	1.09
Physical abuse by parents	0.04	0.06	0.45	0.503	1.04	0.92	1.18
Witnessing Violence Towards Parents	−0.03	0.05	0.32	0.570	0.97	0.87	1.08
Witnessing Violence To Siblings	0.12	0.08	2.43	0.119	1.13	0.97	1.32
Emotional abuse by peers	−0.01	0.05	0.01	0.920	0.99	0.90	1.10
Physical abuse by peers	0.06	0.08	0.61	0.435	1.07	0.91	1.25
Sexual abuse	0.19	0.07	7.39	**0.007**	1.21	1.05	1.38
Constant	−1.60	0.35	20.45	< 0.001	0.20		

*Comment.* Bolded rows indicate statistical significance at *p* < 0.05 level.

Source: own study.

Similar analyses were carried out for the model explaining the incidence of depression based on multiplicity for types of trauma (Table 3). The model was fit to the data χ2(10) = 19.14; *p* = 0.039 and explained a total of 6.1% of the variation in the group, (R^2^_Cox and Snell_ = 0.061). The analysis showed that an increase in multiplicity for physical abuse by peers increased the odds of depression by more than 3 times (*OR* = 3.40), and the presence of multiplicity for sexual abuse increased the risk of depression by almost 2 times (*OR* = 1.98). The other variables in the model were not significantly associated with the occurrence of the disorder.

**Table 3:** Logistic regression coefficients for a model explaining the incidence of depression based on multiplicity for the type of early childhood trauma.

Multiplicity	B	SE	Wald	*p*	OR	95% CI for OR	
						LL	UL
MULTI_PVA	0.06	0.39	0.02	0.89	1.06	0.49	2.28
MULTI_EN	0.82	0.45	3.25	0.07	2.26	0.93	5.48
MULTI_PN	−0.23	0.37	0.37	0.54	0.80	0.38	1.66
MULTI_PNVEA	−0.40	0.37	1.13	0.29	0.67	0.32	1.40
MULTI_PPA	−0.15	0.34	0.20	0.66	0.86	0.44	1.68
MULTI_WITP	−0.37	0.41	0.81	0.37	0.69	0.31	1.54
MULTI_WITS	0.37	0.42	0.76	0.38	1.45	0.63	3.32
MULTI_PEERE	−0.56	0.34	2.72	0.10	0.57	0.29	1.11
Multi_PEERP	1.22	0.45	7.36	**0.01**	3.40	1.40	8.24
MULTI_SEXA	0.68	0.33	4.24	**0.04**	1.98	1.03	3.80
Constant	−1.57	0.33	22.66	< 0.00	0.21		

*Comment.* Bolded rows indicate statistical significance at *p* < 0.05 level.

Source: own study.

Additional univariate models for the MACE total score, Multiplicity and Duration of trauma are shown in Table 4. The analysis showed that as MACE sum by duration increased, the odds of depression increased by 3%. The other parameters were not significantly associated with the occurrence of the disorder.

**Table 4:** Logistic regression coefficients for a model explaining the incidence of depression for parameters of early childhood trauma.

	B	SE	Wald	*P*	OR	95% CI for OR		
						LL	UL	*R* ^2^_Cox and Snell_
MACE - overall score	0.01	0.01	3.29	0.07	1.01	0.99	1.03	0.011
Multiplicity	0.08	0.05	2.31	0.13	1.08	0.98	1.19	0.008
MACE_Sum_by_Duration	0.03	0.01	4.00	0.05	1.03	1.00	1.05	0.013
MACE_Duration	0.01	0.02	0.78	0.38	1.02	0.98	1.06	0.003

Source: own study.

### *Mediating role of maladaptive perfectionism for the relationship between type of trauma and onset of depression*

In the mediation analysis, we conducted tests for the mediating influence of the mediator variable, based on the classical mediation model proposed by Baron and Kenny^83^. According to this model, mediation occurs when the mediator variable (in this case, maladaptive perfectionism) explains part (or all) of the relationship between the independent variable (childhood trauma) and the dependent variable (depression). This model serves as the foundation for many contemporary mediation analyses and is widely used in psychology and social sciences, where researchers aim to explain the mechanisms underlying the observed relationships between variables. According to Baron and Kenny’s assumptions, several conditions must be met to observe mediation, such as statistical significance of the relationship between the independent variable and the mediator, between the mediator and the dependent variable, and a reduction in the strength of the relationship between the independent and dependent variables after accounting for the mediator.

Table 5 shows the results of the analyses of the models considering the mediating role of maladaptive perfectionism between the type of trauma and the occurrence of depression. The analysis conducted showed a mediating effect of perfectionism for all relationships between the type of trauma and the occurrence of depression apart from sexual abuse (the indirect effect was found to be insignificant). The relationship between type of trauma and the occurrence of depression without controlling perfectionism was shown to be significant only for PN, PEERP and SEXA. In most of the models analysed, taking into account the role of the mediating variable did not change the relationship between the type of trauma and the occurrence of the disorder, despite a significant indirect effect. It can therefore be assumed that the relationship between the type of trauma and the occurrence of depression is mediated indirectly through the severity of maladaptive perfectionism.

For physical neglect (PN), a total mediation effect was noted – after accounting for the mediating variable, the relationship between PN and depression was statistically insignificant. Physical neglect through increased maladaptive perfectionism increased the odds of the disorder. The indirect effect accounted for 23% of the combined effect of the relationship between neglect and depression.

For physical abuse by peers (PEERP), the effect was analogous **–** after perfectionism was taken into account in the model, the relationship between PEERP and depression was insignificant. Physical abuse through increased maladaptive perfectionism increased the odds of depression. The indirect effect accounted for 31% of the total effect.

**Table 5:** Models of the mediating role of maladaptive perfectionism for the relationship between type of trauma and occurrence of depression.

Model	Effect^a^	*B*	*SE*	*t/Z*	*p*	CI 95%	
						*LL*	*UL*
Model 1 – PVA	A	0.12	0.02	5.74	< 0.00	0.08	0.16
	B	0.32	0.11	2.96	0.00	0.11	0.53
	C	0.01	0.04	0.16	0.87	−0.06	0.08
	c’	−0.03	0.04	−0.65	0.52	−0.10	0.05
	c – c’	0.04	0.02			**0.01**	**0.07**
Model 2 – EN	A	0.12	0.03	4.38	< 0.00	0.07	0.18
	B	0.27	0.10	2.57	0.01	0.06	0.47
	C	0.07	0.05	1.49	0.14	−0.02	0.17
	c’	0.05	0.05	1.03	0.30	−0.05	0.16
	c - c’	0.03	0.02			**0.01**	**0.07**
Model 3 – PN	A	0.09	0.03	2.70	0.01	0.03	0.16
	B	0.27	0.10	2.61	0.01	0.07	0.47
	C	0.13	0.06	2.20	0.09	0.01	0.25
	c’	0.12	0.06	1.99	0.05	0.01	0.24
	c - c’	0.02	0.01			**0.01**	**0.06**
Model 4 – PNVEA	A	0.16	0.03	5.31	< 0.00	0.10	0.22
	B	0.32	0.11	2.98	0.00	0.11	0.52
	C	< 0.01	0.05	0.06	0.95	−0.10	0.11
	c’	−0.04	0.06	−0.66	0.51	0.15	0.07
	c - c’	0.05	0.02			**0.01**	**0.10**
Model 5 – PPA	A	0.06	0.02	2.66	0.01	0.02	0.11
	B	0.28	0.10	2.73	0.01	0.08	0.48
	C	0.06	0.04	1.49	0.14	−0.02	0.14
	c’	0.05	0.04	1.15	0.25	−0.03	0.13
	c - c’	0.02	0.01			**0.01**	**0.04**
Model 6 – WITP	A	0.08	0.02	3.55	< 0.00	0.04	0.13
	B	0.29	0.10	2.85	0.00	0.09	0.49
	C	0.02	0.04	0.51	0.61	−0.06	0.10
	c’	< 0.01	0.04	0.10	0.99	−0.07	0.08
	c - c’	0.02	0.01			**0.01**	**0.05**
Model 7 – WITS	A	0.10	0.04	2.73	0.01	0.03	0.17
	B	0.27	0.10	2.63	0.01	0.07	0.47
	C	0.11	0.06	1.94	0.05	< −0.01	0.22
	c’	0.09	0.06	1.58	0.11	−0.02	0.21
	c - c’	0.03	0.02			**0.01**	**0.06**
Model 8 – PEERE	A	0.12	0.02	5.56	< 0.00	0.08	0.16
	B	0.30	0.11	2.84	0.00	0.09	0.51
	C	0.03	0.04	0.71	0.48	−0.05	0.10
	c’	−0.01	0.04	−0.21	0.81	−0.09	0.07
	c - c’	0.04	0.02			**0.01**	**0.07**
Model 9 – PEERP	A	0.14	0.04	3.86	< 0.00	0.07	0.21
	B	0.25	0.10	2.47	0.01	0.05	0.46
	C	0.13	0.06	2.32	0.02	0.02	0.24
	c’	0.11	0.06	1.81	0.07	−0.01	0.22
	c - c’	0.04	0.02			**0.01**	**0.08**
Model 10 – SEXA	A	0.18	0.04	5.11	< 0.00	0.11	0.26
	B	0.22	0.11	2.09	0.04	0.01	0.43
	C	0.19	0.06	3.25	0.00	0.07	0.30
	c’	0.15	0.06	2.53	0.01	0.03	0.27
	c - c’	0.04	0.02			−0.01	0.09
Model 11 – MACE SUMA	A	0.02	0.01	6.37	< 0.00	0.02	0.03
	B	0.25	0.11	2.36	0.02	0.04	0.46
	C	0.01	0.01	1.81	0.07	< −0.01	0.03
	c’	0.01	0.01	1.07	0.28	−0.01	0.02
	c - c’	0.01	< 0.01			**< 0.01**	**0.01**

*Comment*. The independent variable is given next to the model (types of early childhood trauma). In each model, the explanatory variable is the occurrence of depression. The mediating variable in the models is maladaptive perfectionism.

^a^ c – c’ is an indirect effect, testing the mediating effect; if the confidence intervals do not contain the value “0” the test result is statistically significant at the *p* < 0.05 level (bold rows).

Source: own study.

### *Mediating role of maladaptive perfectionism for the relationship between multiplicity for types of trauma and the onset of depression*

Table 6 shows the results of the mediation analysis of maladaptive perfectionism for the relationship between multiplicity of trauma types and depression. The analysis showed that almost all mediating effects (except the model for PN) were statistically significant. However, the relationship between multiplicity and the occurrence of the disorder, despite a significant mediating effect, remained statistically insignificant for most cases.

A significant change was observed for 2 models -PEERP and SEXA. For peer physical abuse multiplicity (PEERP), partial mediation was noted – when perfectionism was taken into account in the model, the relationship between PEERP multiplicity and depression decreased, but remained statistically significant. Multiplicity for physical abuse through increased maladaptive perfectionism increased the odds of the disorder. The indirect effect accounted for 21% of the total effect.

For the multiplicity of sexual abuse, there was a total mediation effect – once the mediating role of perfectionism was taken into account, the relationship between SEXA and the occurrence of depression was no longer statistically significant. Multiplicity for sexual abuse through increased maladaptive perfectionism increased the odds of depression. The indirect effect accounted for 31% of the total effect.

**Table 6:** Models of the mediating role of maladaptive perfectionism for the relationship between multiplicity for types of trauma and the onset of depression.

Model	Effect^a^	*B*	*SE*	*t/Z*	*p*	CI 95%	
						*LL*	*UL*
Model 1 – MULTI PVA	a	0.72	0.15	4.62	< 0.00	0.41	1.02
	b	0.29	0.10	2.79	0.01	0.09	0.50
	c	0.19	0.27	0.71	0.48	−0.34	0.71
	c’	0.03	0.29	0.10	0.92	−0.53	0.59
	c – c’	0.21	0.10			**0.05**	**0.43**
Model 2 – MULTI EN	a	0.56	0.18	3.10	0.00	0.21	0.92
	b	0.27	0.10	2.65	0.01	0.07	0.47
	c	0.57	0.34	1.68	0.09	−0.10	1.23
	c’	0.47	0.36	1.31	0.19	−0.23	1.17
	c - c’	0.15	0.07			**0.03**	**0.31**
Model 3 - MULTI PN	a	0.31	0.17	1.86	0.06	−0.02	0.64
	b	0.29	0.10	2.84	0.00	0.09	0.49
	c	0.25	0.29	0.88	0.77	−0.31	0.82
	c’	0.18	0.30	0.62	0.53	−0.40	0.77
	c - c’	0.09	0.06			−0.01	0.23
Model 4 – MULTI PNVEA	a	0.76	0.15	4.90	< 0.00	0.45	1.06
	b	0.31	0.10	2.92	0.00	0.10	0.51
	c	0.06	0.27	0.23	0.81	−0.46	0.59
	c’	−0.12	0.29	−0.43	0.67	−0.69	0.44
	c - c’	0.23	0.10			**0.06**	**0.47**
Model 5 - MULTI PPA	a	0.38	0.16	2.35	0.02	0.06	0.69
	b	0.29	0.10	2.86	0.00	0.09	0.49
	c	0.15	0.27	0.54	0.59	−0.38	0.67
	c’	0.07	0.28	0.24	0.81	−0.48	0.62
	c - c’	0.11	0.06			**0.01**	**0.27**
Model 6 - MULTI WITP	a	0.62	0.20	3.04	0.00	0.22	1.02
	b	0.30	0.10	2.91	0.00	0.10	0.50
	c	0.06	0.34	0.17	0.87	−0.61	0.73
	c’	−0.06	0.35	−0.16	0.87	−0.75	0.63
	c - c’	0.18	0.09			**0.04**	**0.38**
Model 7 - MULTI WITS	a	0.69	0.21	3.24	0.00	0.27	1.11
	b	0.28	0.10	2.71	0.01	0.08	0.48
	c	0.43	0.34	1.28	0.20	−0.23	1.09
	c’	0.27	0.35	0.77	0.44	−0.42	0.97
	c - c’	0.19	0.10			**0.03**	**0.43**
Model 8 - MULTI PEERE	a	0.62	0.16	3.85	< 0.00	0.30	0.93
	b	0.33	0.10	3.13	0.00	0.12	0.53
	c	−0.19	0.28	−0.67	0.50	−0.73	0.36
	c’	−0.41	0.30	−1.37	0.17	−0.99	0.17
	c - c’	0.20	0.09			**0.05**	**0.42**
Model 9 - MULTI PEERP	a	0.95	0.25	3.80	< 0.00	0.46	1.45
	b	0.24	0.10	2.34	0.02	0.04	0.45
	c	1.08	0.38	2.87	0.00	0.34	1.81
	c’	0.96	0.39	2.45	0.01	0.19	1.74
	c - c’	0.23	0.13			**0.02**	**0.52**
Model 10 - MULTI SEXA	a	0.77	0.17	4.39	< 0.00	0.42	1.11
	b	0.26	0.10	2.47	0.01	0.05	0.46
	c	0.65	0.29	2.27	0.02	0.09	1.22
	c’	0.45	0.30	1.47	0.14	−0.15	1.04
	c - c’	0.20	0.11			**0.02**	**0.44**
Model 11 – Multiplicity	a	0.18	0.03	6.20	< 0.00	0.12	0.24
	b	0.26	0.11	2.48	0.01	0.05	0.47
	c	0.08	0.05	1.52	0.13	−0.02	0.18
	c’	0.04	0.05	0.71	0.48	−0.07	0.15
	c - c’	0.05	0.02			**0.01**	**0.10**

*Comment*. The independent variable is given next to the model (multiplicity for types of early childhood trauma). In each model, the explanatory variable is the occurrence of depression. The mediating variable in the models is maladaptive perfectionism.

^a^ c – c’ is an indirect effect, testing the mediating effect; if the confidence intervals do not contain the value “0” the test result is statistically significant at the *p* < 0.05 level (bold rows).

Source: own study.

### *Mediating role of adaptive perfectionism for the relationship between type of trauma and onset of depression*

Table 7 shows the results of the analyses of the mediating role of adaptive perfectionism for the relationship between the type of trauma and the occurrence of depression. The analysis did not demonstrate significance of the mediating role of perfectionism.

**Table 7:** Models of the mediating role of adaptive perfectionism for the relationship between type of trauma and occurrence of depression.

Model	Effect^a^	*B*	*SE*	*t/Z*	*p*	CI 95%	
						*LL*	*UL*
Model 1 – PVA	a	0.01	0.02	0.79	0.43	−0.02	0.05
	b	−0.34	0.11	−2.99	0.00	−0.57	−0.12
	c	0.01	0.04	0.03	0.87	−0.06	0.08
	c’	0.02	0.04	0.43	0.67	−0.06	0.09
	c – c’	−0.01	0.01			−0.02	0.01
Model 2 – EN	a	−0.01	0.02	−0.46	0.64	−0.06	0.04
	b	−0.33	0.11	−2.93	0.00	−0.56	−0.11
	c	0.07	0.05	2.21	0.14	−0.02	0.17
	c’	0.09	0.05	1.65	0.10	−0.02	0.19
	c - c’	0.01	0.01			−0.01	0.02
Model 3 – PN	a	−0.03	0.03	−0.98	0.33	−0.09	0.03
	b	−0.33	0.11	−2.87	0.00	−0.56	−0.10
	c	0.13	0.06	2.20	0.03	0.01	0.25
	c’	0.14	0.06	2.25	0.02	0.02	0.26
	c - c’	0.01	0.01			−0.01	0.04
Model 4 – PNVEA	a	0.06	0.03	2.35	0.02	0.01	0.12
	b	−0.35	0.12	−3.03	0.00	−0.57	−0.12
	c	< 0.01	0.05	< 0.01	0.95	−0.10	0.11
	c’	0.03	0.05	0.63	0.53	−0.07	0.14
	c - c’	−0.02	0.01			−0.05	−0.01
Model 5 – PPA	a	< 0.01	0.02	0.07	0.95	−0.04	0.04
	b	−0.34	0.11	−2.98	0.00	−0.56	−0.12
	c	0.06	0.04	2.23	0.14	−0.02	0.14
	c’	0.07	0.04	1.57	0.12	−0.02	0.15
	c - c’	< −0.01	0.01			−0.02	0.02
Model 6 – WITP	a	0.04	0.02	1.96	0.05	< −0.01	0.08
	b	−0.35	0.11	−3.07	0.00	−0.58	−0.13
	c	0.02	0.04	0.26	0.61	−0.06	0.10
	c’	0.04	0.04	1.02	0.31	−0.04	0.12
	c - c’	−0.01	0.01			−0.03	< 0.01
Model 7 – WITS	a	0.04	0.03	1.16	0.25	−0.03	0.10
	b	−0.36	0.12	−3.13	0.00	−0.59	−0.14
	c	0.11	0.06	3.75	0.05	< −0.01	0.22
	c’	0.14	0.06	2.26	0.02	0.02	0.25
	c - c’	−0.01	0.01			−0.04	0.01
Model 8 – PEERE	a	0.04	0.02	1.99	0.05	< 0.01	0.08
	b	−0.35	0.12	−3.07	0.00	−0.58	−0.13
	c	0.03	0.04	0.50	0.48	−0.05	0.10
	c’	0.04	0.04	1.06	0.29	−0.03	0.12
	c - c’	−0.01	0.01			−0.03	< 0.01
Model 9 – PEERP	a	0.02	0.03	0.73	0.46	−0.04	0.09
	b	−0.36	0.12	−3.09	0.00	−0.59	−0.13
	c	0.13	0.06	5.37	0.02	0.02	0.24
	c’	0.15	0.06	2.60	0.01	0.04	0.27
	c - c’	−0.01	0.01			−0.04	0.02
Model 10 – SEXA	a	0.01	0.03	0.21	0.84	−0.06	0.07
	b	−0.35	0.12	−3.03	0.00	−0.58	−0.12
	c	0.19	0.06	10.55	0.00	0.07	0.30
	c’	0.20	0.06	3.28	0.00	0.08	0.32
	c - c’	−0.01	0.01			−0.03	0.02
Model 11 – MACE SUMA	a	0.01	0.01	1.21	0.23	−0.03	0.01
	b	−0.36	0.11	−3.12	0.00	−0.59	−0.13
	c	0.01	0.01	1.81	0.07	< −0.01	0.03
	c’	0.02	0.01	2.23	0.03	0.01	0.03
	c - c’	< −0.01	< 0.01			−0.01	< 0.01

*Comment*. The independent variable is given next to the model (types of early childhood trauma). In each model, the explanatory variable is the occurrence of depression. The mediating variable in the models is maladaptive perfectionism.

^a^ c – c’ is an indirect effect, testing the mediating effect; if the confidence intervals do not contain the value “0” the test result is statistically significant at the *p* < 0.05 level.

Source: own study.

### *Mediating role of adaptive perfectionism for the relationship between multiplicity for types of trauma and the onset of depression*

Table 8 shows the results of the mediation analysis of maladaptive perfectionism for the relationship between multiplicity of trauma types and depression. The mediating role of adaptive perfectionism was not confirmed - all the mediating effects analysed were found to be statistically insignificant.

**Table 8:** Models of the mediating role of adaptive perfectionism for the relationship between multiplicity for types of trauma and the onset of depression.

Model	Effect^a^	*B*	*SE*	*t/Z*	*p*	CI 95%	
						*LL*	*UL*
Model 1 – MULTI PVA	a	−0.03	0.14	−0.20	0.84	−0.30	0.24
	b	−0.34	0.11	−2.96	0.00	−0.56	−0.11
	c	0.19	0.27	0.50	0.48	−0.34	0.71
	c’	0.23	0.28	0.83	0.41	−0.31	0.77
	c – c’	0.01	0.05			−0.09	0.12
Model 2 – MULTI EN	a	0.02	0.16	0.15	0.88	−0.29	0.34
	b	−0.34	0.11	−2.98	0.00	−0.56	−0.12
	c	0.57	0.34	2.81	0.09	−0.10	1.23
	c’	0.63	0.35	1.78	0.07	−0.06	1.32
	c - c’	−0.01	0.05			−0.13	0.10
Model 3 - MULTI PN	a	−0.10	0.14	−0.72	0.47	−0.39	0.18
	b	−0.34	0.11	−2.94	0.00	−0.56	−0.11
	c	0.25	0.29	0.88	0.77	−0.31	0.82
	c’	0.23	0.30	0.78	0.43	−0.35	0.82
	c - c’	0.03	0.05			−0.06	0.15
Model 4 – MULTI PNVEA	a	0.23	0.14	1.68	0.09	−0.04	0.50
	b	−0.35	0.11	−3.03	0.00	−0.57	−0.12
	c	0.06	0.27	0.05	0.81	−0.46	0.59
	c’	0.19	0.28	0.68	0.50	−0.36	0.74
	c - c’	−0.08	0.06			−0.21	0.02
Model 5 - MULTI PPA	a	0.03	0.14	0.20	0.84	−0.25	0.30
	b	−0.34	0.11	−2.99	0.00	−0.57	−0.12
	c	0.15	0.27	0.29	0.59	−0.38	0.67
	c’	0.19	0.28	0.67	0.50	0.36	0.73
	c - c’	0.01	0.05			−0.11	0.10
Model 6 - MULTI WITP	a	0.35	0.18	2.00	0.05	0.01	0.70
	b	−0.35	0.11	−3.04	0.00	−0.57	−0.12
	c	0.06	0.34	0.03	0.87	−0.61	0.73
	c’	0.25	0.35	0.70	0.48	−0.44	0.94
	c - c’	−0.12	0.08			**−0.30**	**−0.01**
Model 7 - MULTI WITS	a	0.26	0.19	1.40	0.16	−0.11	0.63
	b	−0.36	0.11	−3.10	0.00	−0.58	−0.13
	c	0.43	0.34	1.63	0.20	−0.23	1.09
	c’	0.56	0.35	1.58	0.11	−0.13	1.26
	c - c’	−0.09	0.08			−0.26	0.05
Model 8 - MULTI PEERE	a	0.36	0.14	2.58	0.01	0.08	0.64
	b	−0.34	0.11	−2.91	0.00	−0.56	−0.11
	c	−0.19	0.28	0.45	0.50	−0.73	0.36
	c’	−0.07	0.29	−0.25	0.81	−0.64	0.50
	c - c’	−0.12	0.06			**−0.27**	**−0.02**
Model 9 - MULTI PEERP	a	0.16	0.22	0.73	0.46	−0.27	0.60
	b	−0.37	0.12	−3.13	0.00	−0.60	−0.14
	c	1.08	0.38	8.24	0.00	0.34	1.81
	c’	1.28	0.39	3.24	0.00	0.51	2.05
	c - c’	−0.06	0.10			−0.29	0.13
Model 10 - MULTI SEXA	a	0.25	0.15	1.61	0.11	−0.05	0.56
	b	−0.37	0.12	−3.20	0.00	−0.60	−0.14
	c	0.65	0.29	5.16	0.02	0.09	1.22
	c’	0.75	0.30	2.50	0.01	0.16	1.34
	c - c’	−0.09	0.07			−0.23	0.03
Model 11 – Multiplicity	a	0.04	0.03	1.52	0.13	−0.01	0.09
	b	−0.36	0.11	−3.15	0.00	−0.59	−0.14
	c	0.08	0.05	1.52	0.13	−0.02	0.18
	c’	0.10	0.05	1.94	0.05	< −0.01	0.21
	c - c’	−0.01	0.01			−0.04	0.01

*Comment*. The independent variable is given next to the model (multiplicity for types of early childhood trauma). In each model, the explanatory variable is the occurrence of depression. The mediating variable in the models is adaptive perfectionism.

^a^ c – c’ is an indirect effect, testing the mediating effect; if the confidence intervals do not contain the value “0” the test result is statistically significant at the *p* < 0.05 level.

Source: own study.

### Annex

**Table 9:** Descriptive statistics for parameters related to trauma and perfectionism in the study groups.

Variable	*N*	M	Mdn	SD	Min	Max	Sk.	Kurt.
**Non-clinical group**								
Verbal abuse by parents	235	4.44	4.00	3.60	0.00	10.00	0.12	−1.41
Emotional neglect	235	4.55	4.44	2.81	0.00	10.00	−0.12	−0.89
Physical neglect	235	4.42	4.29	2.17	0.00	10.00	0.06	−0.52
Non-verbal and emotional abuse by parents	235	2.79	1.67	2.54	0.00	8.33	0.54	−0.86
Physical abuse by parents	235	2.99	2.00	3.07	0.00	10.00	0.71	−0.62
Witnessing Violence Towards Parents	235	2.88	0.00	3.48	0.00	10.00	0.83	−0.65
Witnessing Violence To Siblings	233	1.05	0.00	1.97	0.00	8.00	1.89	2.60
Emotional abuse by peers	235	3.69	2.00	3.57	0.00	10.00	0.37	−1.34
Physical abuse by peers	235	0.96	0.00	2.01	0.00	10.00	2.52	6.51
Sexual abuse	235	0.74	0.00	1.72	0.00	10.00	3.10	10.89
Overall MACE score	233	28.58	26.81	17.86	1.43	83.56	0.58	−0.13
MULTI_PVA	235	0.46	0.00	0.50	0.00	1.00	0.16	−1.99
MULTI_EN	235	0.72	1.00	0.45	0.00	1.00	−1.01	−1.00
MULTI_PN	235	0.64	1.00	0.48	0.00	1.00	−0.60	−1.66
MULTI_PNVEA	235	0.46	0.00	0.50	0.00	1.00	0.15	−2.00
MULTI_PPA	235	0.43	0.00	0.50	0.00	1.00	0.29	−1.94
MULTI_WITP	235	0.18	0.00	0.39	0.00	1.00	1.65	0.73
MULTI_WITS	233	0.15	0.00	0.36	0.00	1.00	1.92	1.72
MULTI_PEERE	235	0.40	0.00	0.49	0.00	1.00	0.41	−1.85
Multi_PEERP	235	0.08	0.00	0.27	0.00	1.00	3.09	7.64
MULTI_SEXA	235	0.23	0.00	0.42	0.00	1.00	1.26	−0.40
MACE_MULTI	233	3.78	4.00	2.50	0.00	10.00	0.38	−0.63
Summary score by duration	234	12.70	10.48	9.18	0.32	43.26	1.16	1.19
Training duration	234	12.33	16.00	6.72	0.00	18.00	−0.73	−1.03
Maladaptive perfectionism	231	3.52	3.50	1.33	1.00	6.83	0.24	−0.63
Adaptive perfectionism	231	4.64	4.77	1.14	1.00	7.00	−0.41	0.22
**Clinical group**								
Verbal abuse by parents	73	4.52	6.00	3.93	0.00	10.00	0.10	−1.58
Emotional neglect	73	5.10	5.56	2.55	0.00	10.00	−0.32	−0.27
Physical neglect	73	5.11	5.71	2.64	0.00	10.00	0.03	−0.50
Non-verbal and emotional abuse by parents	73	2.81	1.67	2.75	0.00	10.00	0.66	−0.63
Physical abuse by parents	73	3.64	2.00	3.85	0.00	10.00	0.55	−1.25
Witnessing Violence Towards Parents	73	3.12	2.50	3.33	0.00	10.00	0.58	−0.93
Witnessing Violence To Siblings	73	1.62	0.00	2.66	0.00	10.00	1.49	1.00
Emotional abuse by peers	73	4.03	4.00	3.41	0.00	10.00	0.32	−1.18
Physical abuse by peers	73	1.64	0.00	2.54	0.00	7.50	1.31	0.34
Sexual abuse	73	1.71	0.00	2.92	0.00	10.00	1.71	1.78
Overall MACE score	73	33.30	28.05	23.00	0.00	83.50	0.57	−0.77
MULTI_PVA	73	0.51	1.00	0.50	0.00	1.00	−0.03	−2.06
MULTI_EN	73	0.82	1.00	0.39	0.00	1.00	−1.72	0.98
MULTI_PN	73	0.69	1.00	0.46	0.00	1.00	−0.88	−1.25
MULTI_PNVEA	73	0.48	0.00	0.50	0.00	1.00	0.08	−2.05
MULTI_PPA	73	0.47	0.00	0.50	0.00	1.00	0.14	−2.04
MULTI_WITP	73	0.19	0.00	0.40	0.00	1.00	1.60	0.57
MULTI_WITS	73	0.22	0.00	0.42	0.00	1.00	1.39	−0.08
MULTI_PEERE	73	0.36	0.00	0.48	0.00	1.00	0.61	−1.67
MULTI PEERP	73	0.21	0.00	0.41	0.00	1.00	1.49	0.22
MULTI_SEXA	73	0.37	0.00	0.49	0.00	1.00	0.55	−1.75
MACE_MULTI	73	4.31	4.00	2.93	0.00	10.00	0.32	−1.05
Summary score by duration	73	15.35	11.35	11.41	0.00	46.08	0.94	−0.01
Training duration	73	13.12	17.00	6.51	0.00	18.00	−1.01	−0.55
Maladaptive perfectionism	70	4.08	4.27	1.51	1.00	6.82	−0.25	−0.57
Adaptive perfectionism	70	4.14	4.15	1.34	1.00	6.92	−0.29	−0.27

Source: own study.

## Discussion

In recent years, there has been growing research interest in the impact of early childhood trauma (CT) on the emergence of psychopathology in later stages of human development^84^. Numerous studies indicate that the experience of childhood trauma can result in long-term consequences in the form of emotional and cognitive disorders, including a tendency towards maladaptive perfectionism and an increased risk of depression^27,85,86^. Maladaptive perfectionism, understood as a tendency to set unrealistic expectations and harsh self-evaluation, is increasingly seen as an important mechanism in the aetiology and maintenance of depressive symptoms in adulthood^74,86^.

The main aim of the study was to investigate whether the mechanism of perfectionism significantly mediates the relationship between exposure to early childhood trauma and the development of depression in later stages of life.

### Experience of early childhood trauma and perfectionism in people with depression

According to the results, the group with depression was characterised by significantly higher levels of peer physical abuse (PEERP) and sexual abuse (SEXA) before the age of 18 compared to the healthy group. While both of these forms of violence may involve prolonged exposure of the child or adolescent to the perpetrator, this is not always the case—particularly in instances of sexual abuse. Nevertheless, such exposure, when it occurs, can significantly increase the likelihood of mental disorders in later stages of development^9^. The young person loses trust in people, begins to perceive the world as threatening and uses withdrawal or avoidance mechanisms, which can eventually lead to disrupted relationships and social isolation. Peer or sexual abuse violates intimacy and disrupts a child’s identity development, inducing simultaneous feelings of guilt and shame for the situation and a loss of control over their own life, which can create a risk of developing depression at later stages of life^47,48^. According to biological developmental models, trauma-induced chronic activation of the HPA axis can lead to sustained secretion of stress hormones, which can further contribute to the development of psychopathology, including depression^20^. The results corroborate previous studies in which physical peer violence and sexual abuse in childhood are significant factors in the development of depression in adulthood^27,48,87^.

There were no other statistically significant differences between the study groups in the severity of the different types of trauma. The lack of a direct link between other types of trauma and depression in adulthood may be evidence that the occurrence of abuse or violence in childhood does not directly condition psychopathology in adulthood, but is only a risk factor for it. Depending on the presence or lack of specific mediating factors and specific mechanisms during development and adulthood, depression may develop (only under specific conditions). This is in line with the principle of equipotentiality in developmental psychopathology, which indicates that individuals with initially similar developmental conditions can achieve different developmental outcomes^87^. More recent studies suggest the need to look for mediators between early childhood trauma and depression, including a study by Qin et al.^53^.

The author’s own analyses of the relationship between perfectionism and the occurrence of depression confirm previous research in which maladaptive perfectionism, which arouses many negative feelings and thoughts and lowers self-esteem, is higher in people with depression compared to healthy individuals^58,59^.

### Experience of early childhood trauma in people with depression - mediating role of perfectionism

The results of this study show that maladaptive perfectionism is a mediating factor between the occurrence of early childhood trauma (CT) in the form of physical neglect (PN), physical abuse by peers (PEERP) and sexual abuse (SEXA) and depression in adulthood. These findings fit with both the PSDM model^62^ and the SRMP model^63^, where CT may be a factor favouring the development of perfectionism, which should help satisfy basic emotional needs, promote a sense of control and reduce feelings of shame and helplessness^62,63^.

Maladaptive perfectionism, which may develop as a coping mechanism in response to childhood physical neglect (PN), is thought to be linked to chronic stress through biological mechanisms. According to the biological model, this maladaptive perfectionism could activate the hypothalamic-pituitary-adrenal (HPA) axis, leading to prolonged cortisol secretion. Chronic exposure to elevated cortisol levels is associated with the dysregulation of stress response systems and has been linked to various mental health issues, including depression and anxiety. According to Carver and Scheider^21^, poor self-regulation resulting from the nature of a person’s set goal (e.g. when implicit goals hinder the achievement of consciously chosen goals) can cause a person with perfectionistic behaviour to continually strive to achieve standards that are impossible to meet, leading to emotional exhaustion, frustration and a sense of failure. Low self-esteem and a strong need for approval exacerbate the risk of depression, especially when perfectionism becomes an important tool for coping with difficult emotions arising from neglect^86^. A child who experiences physical neglect in childhood, including lack of food or adequate clothing, neglect in the area of medical and/or educational support, often feels a strong sense of shame and inferiority, as well as a lack of trust in other people. This is because such an individual is aware that people in their immediate environment that they are growing up in can see what is going on and do not respond to their needs, which often leads to isolation and withdrawal from both social relationships and educational and developmental activities. An explanation for the total mediation effect found in this study when taking into account the mediating variable of perfectionism can also be sought in attachment theory^89^. A person who, through neglect, experienced insecure attachment as a child may develop perfectionism as a way of satisfying the need for acceptance and love. Having high expectations towards oneself becomes a way to gain approval, but this leads to chronic dissatisfaction as such a person never feels that they are good enough. This can also lead to depression resulting from a sense of rejection. In a study by Fang and Wang^87^, perfectionism plays a partial mediating role in the relationship between the close-dependent composite dimension, anxiety dimension of attachment and depression, which partially supports the effect obtained in this study.

Further results obtained in the present study indicate that the development of depression in adulthood through perfectionism also occurs in people who experienced sexual abuse in childhood. The authors of earlier studies^90^ and of this article highlight the importance of perfectionism as a compensation strategy. As in the case of physical neglect, sexual abuse can develop deep feelings of shame, guilt and worthlessness in a child, also inducing a sense of responsibility for what has happened to them and a loss of trust in people. Extremely low self-esteem develops, associated with excessive self-criticism. Therefore, the development of perfectionism in a sexually abused child may represent a kind of adaptive response to the trauma, as the child tries to be ‘perfect’ and take control of their life in order to reduce further suffering, shame and a sense of rejection by others. However, the inability to cope with the expectations set for oneself exacerbates feelings of worthlessness, is combined with constant feelings of stress and helplessness and lack of self-acceptance, leading to depression^72,74^. Victims of sexual abuse may also avoid close relationships with others, which leads to a deepening sense of loneliness and depressive symptoms. Perfectionism can also be a way of regulating emotions by distracting from difficult emotions related to the trauma of childhood sexual abuse, but in the long term, avoidance of negative emotions usually leads to the development of psychopathology, including depressive disorders^91,92^.

The abovementioned patterns of perfectionism can also be analysed in the context of the correlation of peer physical violence experienced in childhood with the occurrence of depression in adulthood, which is supported by the results of this study, indicating an overall mediation effect. An explanation of the role of perfectionism in the development of depression in individuals with peer trauma should be further completed by reinforcement and punishment theory^93^, which claims that perfectionism may represent a learned response to reinforcements or punishments received in childhood. A child rewarded by peers for excellence and physically punished for not being good enough may develop maladaptive perfectionism to avoid abuse from them. This negative reinforcement makes perfectionism a coping strategy in the form of overcompensation, at the same time contributing to excessive stress. Physical peer violence often leads to the development of a belief that a person is weak and unable to cope, which in the long term can contribute to the development and maintenance of depression.

The results of this study did not reveal a mediating role of adaptive perfectionism in the relationships studied – it neither had a protective function nor was it a risk factor for the development of depression. Adaptive perfectionism, defined as a set of traits that help an individual achieve goals and develop a positive self-image, is considered to be less harmful to a person’s mental health than maladaptive perfectionism^88^. It is not associated with chronic stress, a sense of failure or excessive self-criticism^92^, but with better coping and goal achievement, which can reduce vulnerability to depression. CT significantly affects the psyche and cognitive patterns of the developing child. As highlighted earlier, it often leads to negative patterns of thinking about oneself and the world and interpreting reality, which exacerbate depressive symptoms despite having perfectionist traits, as indicated by many studies^25–27,93^. Therefore, adaptive perfectionism in individuals with trauma may help them cope with difficulties, but at the same time may not be a powerful enough mechanism to counterbalance the long-term psychological effects of CT, which are deeply rooted in cerebral cognitive and emotional structures^7,94^ and its influence may not be significant enough as to be able to counteract the developing depression. As a result, trauma and depression may or may not have a direct relationship, and adaptive perfectionism is not a strong enough mechanism to influence this relationship.

In conclusion, the results of the present study confirm the need to look for mechanisms that contribute to depression in people experiencing early childhood trauma. As shown above, one such mechanism is maladaptive perfectionism, which is an important risk factor for the development of depression in people who experienced physical peer violence, physical neglect and sexual abuse in childhood. Statistical analyses did not reveal associations of other types of trauma with depression in adulthood or the presence of mediating perfectionism. An important finding is the lack of association of adaptive perfectionism with CT and the occurrence of depression in adulthood, which leads us to reflect that the mechanism is too weak to alter the pathway between trauma and depression.

### Clinical implications of the study results

The results of this study have significant clinical implications, particularly in the diagnosis, treatment, and prevention of depression. The findings highlight the crucial role of childhood traumatic experiences, including physical neglect and sexual abuse, in the increased risk of depression in later life. Specifically, individuals with a history of physical or sexual abuse demonstrate higher levels of depressive symptoms, which suggests the need for early diagnosis and intervention in individuals with such traumatic experiences. Psychiatrists and psychotherapists could apply these findings to identify individuals particularly at risk of depression, as well as to develop targeted therapies that consider childhood trauma as a key element in patient treatment.

Another important aspect of the study is the role of maladaptive perfectionism as a mediator in the relationship between trauma and depression. The results indicate that maladaptive perfectionism, developed as a coping mechanism in response to trauma, can significantly contribute to the exacerbation of depressive symptoms. Therefore, in clinical practice, this factor should be considered in the treatment of individuals with depression. Specifically, cognitive-behavioural therapy (CBT) could focus on modifying perfectionistic beliefs and attitudes, which may help improve patients’ well-being and reduce the risk of developing depression.

Furthermore, the study results underline the necessity for the implementation of early intervention programmes that focus on children and adolescents experiencing traumatic events, such as neglect or abuse. Interventions aimed at improving coping strategies for trauma and promoting mental health in younger populations can have a long-term impact on reducing the risk of depression in adulthood. Working with families, schools, and healthcare institutions is crucial for identifying and responding to mental health issues in children, which could help prevent later disorders, such as depression.

In conclusion, the findings of this study provide valuable insights into more effective methods of diagnosing, treating, and preventing depression. Applying these results in clinical practice could contribute to the development of more precise therapeutic strategies that take into account both individual traumatic experiences and defence mechanisms, such as maladaptive perfectionism.

### Limitations

While these analyses have identified some important relationships, they also have some limitations. The first major limitation is the cross-sectional nature of the study, making it impossible to establish clear causal relationships. Cross-sectional studies make it possible to identify correlations between variables, but cannot determine whether early childhood trauma leads to the development of perfectionism, which then influences the development of depression. The results suggesting such a relationship require further verification through longitudinal studies to better understand changes taking place over time and complex interactions between the analysed variables.

The second limitation is reliance on self-assessment questionnaire tools. This form of research carries the risk of distorting the result due to the subjective perception of the participants’ own experiences and behaviours, which may be affected by memory errors, interpretation or the so-called positive effect among the respondents. These limitations may be particularly apparent when assessing the experience of trauma and the degree of perfectionism, which can be strongly emotionally and contextually influenced. The use of observational methods, clinical interviews and information from third parties (e.g. relatives) could make it possible to obtain more objective data.

Another limitation is insufficient control over other variables that can influence outcomes, such as social support, genetic factors or environmental stress levels. These additional variables may play a role as both mediators and moderators in the relationship between childhood trauma, perfectionism and depression, which could significantly modify the results. For instance, the presence of social support may moderate the impact of trauma on the development of perfectionism^94^ and reduce the risk of depression^95^, but this variable was not taken into consideration in the analysis.

### Future research

In order to more effectively understand the mechanisms of the links between childhood trauma, perfectionism and depression, future research should be extended to include a longitudinal perspective. This type of research could ensure a better understanding of the development of maladaptive perfectionism as a mechanism emerging in response to traumatic experiences and assess its role as a mediator in the development of depression.

In the future, it would also be worthwhile to analyse the role of moderating and mediating variables such as social support, stress coping strategies and attachment style. Social support from family or friends could act as a buffer against the negative effects of trauma and influence the way individuals develop perfectionism and cope with psychological burdens.

Moreover, future research could use diverse research methods. The use of more objective measurement methods such as clinical interviews, behavioural observation tools or the use of third-party information could minimise the risk of errors resulting from the self-reporting nature of these tools. An alternative could also be to use biological indicators (e.g. cortisol levels as an indicator of stress), which would allow for a more accurate understanding of physiological mechanisms.

An important aspect of future analyses could be to take into account the cultural context. Different cultures define and experience perfectionism and trauma differently, which may affect the understanding and interpretation of the results. For example, the level of social acceptance of certain forms of perfectionism may vary from one country to the next, affecting the associated risk of developing depression. Comparative research across cultures could clarify whether and how cultural context moderates the links between childhood trauma, perfectionism and depression.

## Data Availability

All additional data from this study are available from the authors without restriction upon request. Data available from the author: Magdalena Chęć, email address: magdalena.chec@usz.edu.pl lub Sylwia Michałowska, email adress: sylwia.michalowska@usz.edu.pl.
